# Machine Learning for Predicting the Risk for Childhood Asthma Using Prenatal, Perinatal, Postnatal and Environmental Factors

**DOI:** 10.3390/healthcare9111464

**Published:** 2021-10-29

**Authors:** Zineb Jeddi, Ihsane Gryech, Mounir Ghogho, Maryame EL Hammoumi, Chafiq Mahraoui

**Affiliations:** 1 TICLab, College of Engineering & Architecture, International University of Rabat, Rabat 11103, Morocco; zineb.jeddi@uir.ac.ma; 2 ENSIAS, Mohammed V University in Rabat, Rabat 10000, Morocco; 3 School of IEEE, University of Leeds, Leeds LS2 9JT, UK; 4 Pediatrics Department, CHU, Rabat 10000, Morocco; drmaryamehm@gmail.com (M.E.H.); cmahraoui@gmail.com (C.M.)

**Keywords:** asthma, machine learning, prediction, risk factors, environment, prevention, pediatrics

## Abstract

The prevalence rate for childhood asthma and its associated risk factors vary significantly across countries and regions. In the case of Morocco, the scarcity of available medical data makes scientific research on diseases such as asthma very challenging. In this paper, we build machine learning models to predict the occurrence of childhood asthma using data from a prospective study of 202 children with and without asthma. The association between different factors and asthma diagnosis is first assessed using a Chi-squared test. Then, predictive models such as logistic regression analysis, decision trees, random forest and support vector machine are used to explore the relationship between childhood asthma and the various risk factors. First, data were pre-processed using a Chi-squared feature selection, 19 out of the 36 factors were found to be significantly associated (*p*-value < 0.05) with childhood asthma; these include: history of atopic diseases in the family, presence of mites, cold air, strong odors and mold in the child’s environment, mode of birth, breastfeeding and early life habits and exposures. For asthma prediction, random forest yielded the best predictive performance (accuracy = 84.9%), followed by logistic regression (accuracy = 82.57%), support vector machine (accuracy = 82.5%) and decision trees (accuracy = 75.19%). The decision tree model has the advantage of being easily interpreted. This study identified important maternal and prenatal risk factors for childhood asthma, the majority of which are avoidable. Appropriate steps are needed to raise awareness about the prenatal risk factors.

## 1. Introduction

Asthma is the most common chronic disease among children in the world. It is a multi-factorial disease caused by a chronic inflammation of the airways. This chronic respiratory condition is characterized by several persistent symptoms, including cough, wheeze, dyspnea, and chest tightness. According to the world health organization, asthma affected 262 million people and was responsible for 461,000 deaths worldwide in 2019 [1,2]. Globally, asthma affects approximately 334 million people per year and 14% of the world’s children experience asthma symptoms [3]. Even though the prevalence of childhood asthma varies between countries across the world, studies have shown that asthma prevalence is increasing at a high rate in developing countries [4], especially in densely populated areas [5]. In contrast, many developed countries have managed to slow down the increasing rate of asthma prevalence among their populations [6]. In Morocco, asthma is much more prevalent in children than in adults. The prevalence rate of asthma in children between the ages of 13 and 14 is 20%, whereas for adults, it varies between 15% and 17% [7]. Given the complex nature of this disease, several factors can be responsible for the increasing rate of childhood asthma prevalence, including genetic predisposition factors [4], environmental factors [8], prenatal and postnatal factors as well as the other factors related to the health of the mother during pregnancy and delivery periods. Studies have shown that the mother’s overall health during pregnancy in the prenatal period is significantly associated with developing asthma in the early years of childhood [9]. In fact, studies have shown that maternal diseases during pregnancy such as diabetes, atopic diseases, asthma and hypertension increase the risk of asthma for the child [10]. Moreover, other studies have also shown that forceps-assisted deliveries, maternal smoking during pregnancy, and low birth weight may also present significant risk factors for childhood asthma [10,11,12]. On the other hand, it was shown in [13] that frequent maternal exposure to farm animals during pregnancy can help prevent childhood asthma [14]. In the case of Morocco, the non-availability of medical data due to patients’ privacy and the lack of electronic health records makes scientific research on diseases such as asthma very challenging and limited. However, because of the increasing prevalence of asthma among the pediatric population, focused efforts must be dedicated to providing a better understanding of the disease and thus elaborate better prevention and management strategies for childhood asthma. In this study, we utilize data from the Ibn Sina Hospital Center (CHUIS) to contribute to the assessment of the Asthma situation in Morocco. We first investigate perinatal, prenatal, postnatal and environmental risk factors for asthma, using patient data. We then use machine learning models to predict the occurrence of childhood asthma and to quantify the importance of the identified risk factors. It is worth pointing out that previous studies have focused on statistical methods to infer associations between asthma and risk factors.

## 2. Materials and Methods

**Figure 1:**
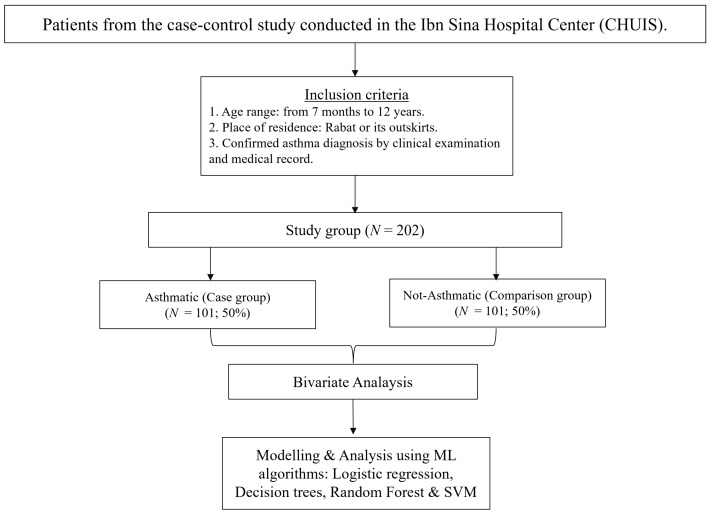
Flow chart of the study.

In this section, we describe the process followed in our study (Figure 1). One of the main goals of this work is to lay the ground for future work on uncovering asthma risk factors in Morocco. Thus, we use a Moroccan data set.

### 2.1. Data Collection

A case-control study of 202 children was previously conducted in the Ibn Sina Hospital Center (CHUIS). A dataset resulted from this study and was made available to us for analysis. The study consists of children with (*N* = 101) and without (*N* = 101) asthma. The data collection was conducted over a period of 4 months, from May to September 2018. The age of the children included in the study varies from 7 months to 12 years. The data collection took place in the pneumology, allergology and infectiology service at the Children’s Hospital in Rabat. The doctors participating in the study interviewed the child’s mother in the local language (Moroccan dialect). The questions used for the interviews were designed by pediatricians to gather information about prenatal, perinatal and postnatal periods, as well as factors that are potentially associated with childhood asthma, including family history, environment, and other exposure features during early childhood (first two years of life). All variables were binary categorical.

### 2.2. Inclusion Criteria

The inclusion criteria used by the medical doctors for data collection are as follows:

- Age range: this ranges from 7 months to 12 years.
- Place of residence: only patients living in the city of Rabat or its outskirts were included in the study.
- Confirmed asthma diagnosis: the diagnosis was based on a clinical examination by a pediatrician who assessed tangible symptoms such as wheezing, chest tightness, difficulty in breathing induced by physical exercise and dry coughs, especially at night.

### 2.3. Data Analysis

Data were analyzed using the R software. First, we started with a primary feature selection using a Chi-squared test. This bivariate analysis allowed to assess the association between the response variable and the other variables in the dataset. Variables associations with *p*-value < 0.05 were considered to be significant risk factors for childhood asthma. For the modeling part, we partitioned the data into two subsets: 80% for training and 20% for testing. Second, we performed logistic regression. We used backward stepwise logistic regression to select the final model where only significant variables (*p*-value < 0.05) were retained in the final model. In order to identify the best model for predicting childhood asthma, we also built predictive models based on Decision Tree and Random forest techniques. Then, we used both the training and the testing data sets to compare the performance of the different models and identify the model that better predicts childhood asthma diagnosis. To evaluate the predictive ability of the different models, we used different performance metrics, namely accuracy, F1 scores, AUC-ROC, sensitivity (the false positive, Sn) and specificity (the false negative, Sp).

## 3. Results

Table 1 displays descriptive characteristics and the association between prenatal, perinatal, postnatal factors and childhood asthma, measured by the Chi-squared test of independence. The history of having maternal atopic tendencies and environmental factors such as cold air, strong odors, reported dust mites, pollen, mold in the child’s environment and having pets (during the prenatal, perinatal and postnatal periods) were all significantly associated with childhood asthma (*p*-values < 0.05). Other significant factors are related to the mother’s state of health, including consumption of “antibiotics/paracetamol” during pregnancy, a cesarean mode of birth, maternal overweight during pregnancy and a paternal age of more than 34 years at the child’s birth. In the postnatal period and early childhood, other features were also significant predictors for asthma; these include breastfeeding, dietary diversity when the child is aged between 4 and 6 months and also when the child is aged over 6 months. Overweight and the use of antibiotics by the child in the first two years were also significantly associated with childhood asthma in the bivariate analysis.

### 3.1. Logistic Regression

Despite its name, logistic regression (LR) is a classification model rather than a regression model. It is an efficient method for binary and linear classification. For a model with two predictors, $x_{1}$ and $x_{2}$, and one binary (Bernoulli) response variable *Y*, the probability for Y=1, denoted as p=P(Y=1), is expressed as

(1) $$p=\frac{1}{1+e^{-(b_{0}+b_{1}x_{1}+b_{2}x_{2})}}$$

where $b_{0}+b_{1}x_{1}+b_{2}x_{2}$ are parameters of the model. LR is the transformation of a linear regression using the Sigmoid function to restrict the value of *p* to be between 0 and 1.

Table 2 displays the multivariate odds ratios (OR) and the confidence intervals (2.5–97.5%) obtained by the statistical analysis of the logistic regression model. Environmental factors, including reported dust mites and the cold airflow in the child’s environment were the most significant factors in predicting childhood asthma. The chances of having asthma were approximately a hundred times higher among children who were born in environments with a reported presence of mites (adjusted OR = 101.23, 95% CI = 13.39–2271.27) and 21 times higher in an environment with a persistent cold airflow (adjusted OR = 21.62, 95% CI = 2.18–335.19). Having family members with cold (adjusted OR = 5.98, 95% CI = 1.32–31.15) and flu (adjusted OR = 11.61, 95% CI = 2.31–76.33) in the environment of the child during the neonatal period also increases the chances of childhood asthma. Among mothers who reported having a history of an atopic disease, the odds of having childhood asthma were approximately nineteen-fold higher (adjusted OR = 19.04, 95% CI = 3.83–126.39). Parents age at birth was also a relevant factor to predict childhood asthma. A maternal age that is above 35 years (adjusted OR = 53.13, 95% CI = 4.24–850.82) or below 25 years (adjusted OR = 7.19, 95% CI = 1.81–33.17) as well as a paternal age that is above 34 years (adjusted OR = 13.50, 95% CI = 2.66–84.79) were found to be highly associated with childhood asthma in this model. The mode of birth was also an important factor in predicting childhood asthma, where the chances of developing asthma were almost seven-fold higher among children who were delivered via a cesarean section (adjusted OR = 6.77, 95% CI = 2.12–25.75). Breastfeeding in the first two years (adjusted OR = 0.03, 95% CI = 0.01–0.12) and diversifying the baby’s diet between 4 and 6 months of age (adjusted OR = 0.35, 95% CI = 0.09–1.24) were found to be protective against childhood asthma.

### 3.2. Decision Tree Model

Decision trees are one of the most popular non-parametric supervised learning methods for classification and regression. The goal of a decision tree is to create a model that predicts a targeted value by learning simple decision rules from the data features. For decision trees, internal nodes denote a test on an attribute, the branch represents an outcome of the test, and the leaf node holds a class label. In our case, we built a decision tree classifier using the features selected based on the Chi-squared test. When training the model, the metric used to perform the splits is the Gini’s Diversity Index (GDI), which is a measure of the node’s impurity. The size of the tree was determined by setting a minimum of 10 observations per leaf node. Each node shows respectively:

- The predicted class (‘Asthma’ or ‘Not asthma’).
- The predicted probability of asthma diagnosis.
- The percentage of observations in the node.

**Figure 2:**
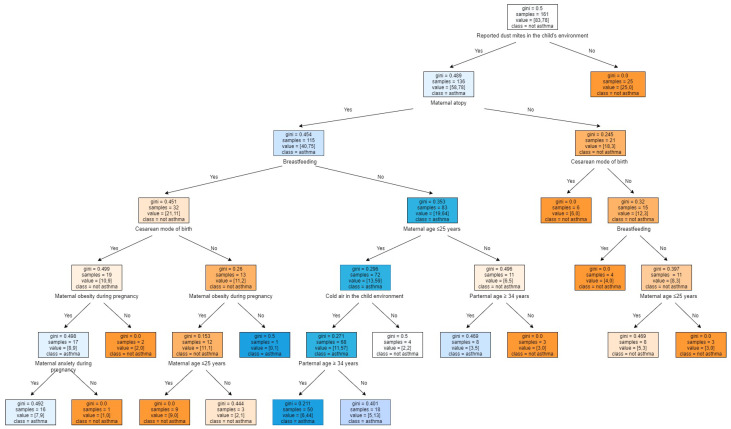
The obtained decision tree model-based classifier.

The decision tree in Figure 2 indicates that the most influential attribute in determining childhood asthma is the reported ‘presence of dust mites in the child’s environment’.

For the decision tree interpretation, the first question asked is ’ are there any reported dust mites in the child’s environment?’. If the answer is yes, the model verifies if the patient’s mother has reported having a history of atopic diseases. If the answer is no, the model verifies if the mother had a cesarean mode of birth, if the answer is now yes, the decision tree classifies the case as non-asthmatic. Similarly, all the tree branches are interpreted in the same manner.

### 3.3. Random Forest Model

Random forest is a very effective ensemble learning technique that combines many classifiers to provide solutions to complex problems. After using decision trees, we decided to use random forest, which consists of many decision trees. The ’forest’ of trees generated by the random forest algorithm is trained through bagging or bootstrap aggregating. Increasing the number of trees increases the precision of the outcome and reduces overfitting. In this work, we used 100 trees to ’grow’ the forest (using a full feature set). The number of features randomly selected to perform each split was set to be the square root of the number of features, which is a typical choice. Since in this study, we have 36 features in total, the number of features that are randomly selected at each node is set to 6 features. The variable importance is computed using the mean decrease in the Gini index. Table 3 shows the 19 most important risk factors associated with childhood asthma.

### 3.4. Support Vector Machine

Support vector machine is a machine learning technique that relies on kernel functions to provide the best fit to observed data [15]. It aims to map a high-dimensional feature space to the considered output. Different kernel functions can be adopted [16]. In this work, we assume a Gaussian kernel function. Hence, the prediction takes in the following form

(2) $$\hat{Y}=\sum_{i=1}^{m}\theta_{i}\exp\left(\frac{-\|X-x_{i}{\|}^{2}}{\gamma }\right),$$

where $X=[X_{1},\ldots X_{p}]$, $x_{i}$ is the value of the feature vector that corresponds to the *i*th observation, *m* is the number of observations, γ is a tuning parameter, and the $\theta_{i}$’s can be computed based on the cost function by evaluating the difference between the predicted values and the real values of pollutants’ concentrations, to a threshold ϵ [17].

Table 4 describes the obtained results when the SVM model is adopted. It is shown that SVM did not bypass logistic regression and random forest but still yielded better results than decision trees.

### 3.5. Comparison of Performance of Models

**Figure 3:**
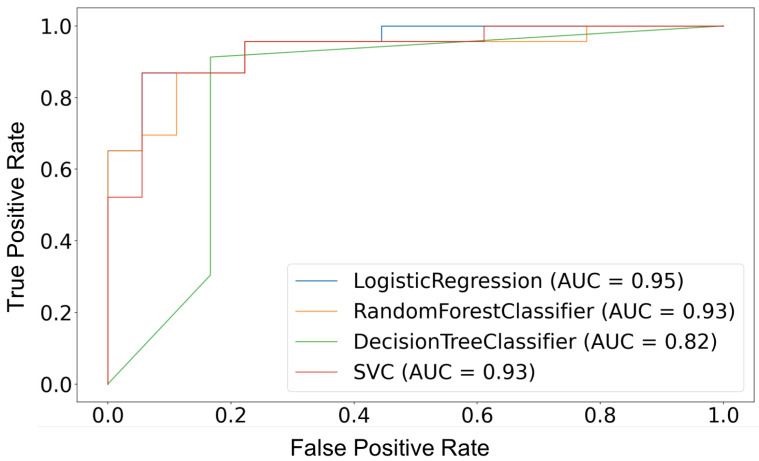
Models’ ROC curve.

In terms of predictive ability, the random forest yielded the best performance. It provided the most accurate results when predicting childhood asthma; it correctly classified 87.8% of the cases when applied to the test data set. The decision tree model has correctly classified 85.3% of the test cases. The decision tree identified “Asthma” cases with 91.30% sensitivity and “Not asthma” cases with 78% specificity. When evaluated on the test data set, the logistic regression model performed with an accuracy of 85.36%, a sensitivity of 83% and a 83% specificity (see Table 4). To settle the ambiguous results of the contest between logistic regression and decision trees. We compute a 10-fold cross validation and F1 scores, and we display an AUC-ROC for each one of our models. The average accuracy for 10-folds cross validation showed that random forest outperformed logistic regression and SVM. On the other hand, decision trees scored the lowest accuracy, but are still helpful in terms of interpretability. Although random forest yielded the best accuracy results, it is evident from the plot in Figure 3 that the AUC for the logistic regression ROC curve is higher than that for random forest and decision trees. This means that logistic regression did a better job of classifying the positive class in the dataset. One may ask why the AUC for logistic regression is better than that of random forest, when random forest “seems” to outperform logistic regression with respect to accuracy. Our answer would be that accuracy is computed at the threshold value of 0.5. While AUC is computed by adding all the “accuracies” computed for all the possible threshold values. ROC can be seen as an average (expected value) of those accuracies when they are computed for all threshold values.

## 4. Discussion

In the present study, we found that environmental factors, prenatal maternal exposures, complications during pregnancy, perinatal and postnatal personal exposures, along with other factors related to parental histories of atopy, can significantly increase the risk of asthma prevalence in pre-schooled children (children under 7 years old). As observed in previous studies [18,19], maternal histories of atopy were associated with an increased risk of childhood asthma. In this study, approximately 23.76% of the interviewed mothers reported having a history of an atopic disease. This study found that parental age at birth is significantly associated with the prevalence of asthma in 7-year-old children. Indeed, a maternal age higher than 35 years or lower than 24 were associated with high risks of childhood asthma, while a paternal age higher than 35 years was also associated with high risks of developing childhood asthma. For instance, 21.78% of asthma cases reported a paternal age under 24 years. In previous studies, young maternal age and young paternal age were found associated with various child outcomes, including asthma prevalence in offspring; our results indicate that also maternal and paternal age of ≥35 years could be risk factors for childhood asthma [20,21,22]. In another study, using data from the Swedish Medical Birth register [23], results have shown that a decreased risk of asthma prevalence in childhood is associated with an increasing paternal age; this result was also confirmed in [22]. The difference in our results may reflect contrasting adverse factors related to behavioral, social and lifestyle agents that can characterize a middle income country such as Morocco [24]. In line with many studies [25,26,27,28], our results indicate that reported environmental factors such as cold airflow, strong odors, reported dust mites, pollen, mold and having pets in the neonatal period are significantly associated with the prevalence of childhood asthma. In this study, approximately 30.69% of asthma cases reported dust mites in their environments, 12.87% reported the presence of pollen in their surroundings, 11.88% reported the presence of mold in their surroundings and 6.93% stated an exposure to strong odors. In addition to these environmental factors, 22.77% of asthma cases reported that at least one family member had a respiratory infection (cold) in the neonatal period. Consuming antibiotics and/or paracetamol during pregnancy also was found to increase the risk of childhood asthma. Different studies provided supporting results; in [29], the authors showed that exposure to antibiotics during pregnancy was significantly associated with a small increased risk of asthma in pre-schooled children [28,29]. Different studies indicate that antibiotic use can have long-term altering effects on the vaginal bacterial flora, which may have adverse impacts on the health outcomes of the child [30,31]. Moreover, we also found that maternal obesity during pregnancy is significantly associated with asthma prevalence in children. Concerning the mode of birth, evidence for the health risks related to the perinatal period is accumulating. Children born via a cesarean delivery are at higher risks of developing autoimmune diseases such as asthma and allergies [32,33,34,35]. Our results also confirm a highly significant association between a cesarean mode of delivery and the increased risks of asthma prevalence. In our study 58.42% of children who developed asthma in their early childhood were born via a cesarean section. Furthermore, early childhood is also considered as a critical period for the occurrence of many risk factors related to environmental exposure and lifestyle habits. Although breastfeeding and delivery mode appear to modify the risk of childhood allergic outcomes, it is unclear whether they have the potential to attenuate or intensify the risk associated with developing asthma in offspring [34]. However, in our study, postnatal factors such as breastfeeding and dietary diversity between 4 months and 6 months old were found to be significantly associated with asthma prevalence among children. For instance, 45.54% of children who developed asthma did not receive maternal breastfeeding, and only 20% of patients had diverse nutrition between 4 months and 6 months old.

There are also some limitations to this study. Since the data set provided to us was obtained from a case-control study, the presence of selection bias and recall bias was a major concern. The study site, Ibn Sina childrens hospital, is an almost free of charge university hospital that cares for the local community coming from Rabat-Salé-Temara agglomeration, which is characterized by major social differences across and within areas. Although unlikely, there is also a possibility that cases and controls from places outside the hospital’s service area may have come to the hospital for care hence resulting in selection bias. The outcome, i.e., child’s asthma status, was determined clinically by the primary care physician, but exposure data were self-reported. Differential recall of exposures by mothers of children with asthma as compared to mothers of children without asthma could result in differential misclassification (recall) bias. Such type of bias is more common in case-control studies of children with severe medical conditions such as birth defects, and hence less likely to have occurred in our study. Furthermore, the study was designed to ask respondents about different types of exposures; thus, it is unlikely for mothers of children with asthma to remember exactly the prenatal exposures. However, to minimize interviewer bias, the researcher who interviewed study participants was blinded to the asthma status of the child. Nonetheless, interviewer bias in face-to-face studies is difficult to eliminate completely. Prenatal exposure to pets was not measured objectively and may have resulted in misclassification errors. Obtaining access to medical data sets is very challenging due to patients’ privacy and the lack of electronic health records. The current study was performed at one regional hospital, where we were only able to obtain access to data of 202 patients. Larger scale studies are needed to improve prediction performance and generalize our results beyond the regional nature of our study.

## 5. Conclusions

The findings of this study emphasize the potential importance of assessing prenatal, perinatal and postnatal risk factors associated with childhood asthma. In order to reduce the risks of developing childhood asthma in our population, the results from this study can provide relevant support for further use when elaborating the right prevention strategies regarding prenatal and during pregnancy care. Moreover, the risk factors identified in this study can help us predict children that are prone to develop asthma in the early stages of life and thereby allow a secure set of interventions that could prevent them from developing the disease and thus help them lead a healthy and normal childhood. 

## Figures and Tables

**Table 1:** Descriptive characteristics and results of Chi-squared test of independence for the study sample.

Characteristics (*n* = 202)	Children with Asthma (*N* = 101, 50%)	Children without Asthma (*N* = 101, 50%)	Chi-Square Test (*p*-Value)
Factors related to family history			
Maternal atopy	28 (84.85%)	5 (15.15%)	1.263 $\times \;10^{-5}$
Paternal atopy	17 (65.38%)	9 (34.62%)	0.09361
History of an atopic disease in brothers or sisters	9 (56.25%)	7 (43.75%)	0.6032
Personal atopic dermatitis	13 (61.90%)	8 (0.3809524)	0.2502
Factors related to the child environment			
Reported dust mites in the child environment	31 (96.87%)	1 (3.13%)	8.089 $\times \;10^{-9}$
Reported pets (cats) in the child environment	7 (77.78%)	2 (22.22%)	0.08897
Reported pollen in the child environment	13 (0.7222222)	5 (27.78%)	0.04875
Reported mold in the child environment	12 (85.71%)	2 (14.20%)	0.005719
Reported cold airflow in the child environment	15 (83.34%)	3 (16.67%)	0.003115
Reported respiratory infections in family members (cold)	23 (76.67%)	7 ( 23.34%)	0.001589
Reported respiratory infections in family members (flu)	15 (62.50%)	9 (37.5%)	0.1931
Reported respiratory infections in family members (sinusitis)	5 (83.34%)	1 (16.67%)	0.09819
Prenatal, Perinatal and postnatal factors			
Maternal age ≤ 25 years	33 (76.75%)	10 (23.26%)	8.027 $\times \;10^{-5}$
Maternal age ≥ 35 years	5 (62.5%)	3 (37.50%)	0.4717
Paternal age ≤ 24 years	(62.50%)	(37.50%)	0.4717
Paternal age ≥ 34 years	22 (%)	7 (%)	0.002679
Maternal obesity during pregnancy	15 (75%)	5 (25%)	0.01878
Maternal anxiety during pregnancy	16 (69.57%)	7 (30.43%)	0.04674
Exposure to secondhand smoking during pregnancy	25 (56.82%)	19 (43.18%)	0.3076
Consumption of antibiotics/paracetamol during pregnancy	9 (90%)	1 (10%)	0.009641
Underweight child	9 (75%)	3 (25%)	0.07483
Overweight child	15 (68.18%)	7 (31.81%)	0.07149
Prematurity	5 (62.50%)	3 (37.50%)	0.4717
Cesarian mode of birth	59 (60.83%)	38 (39.17%)	0.003177
Breastfeeding	55 (38.73%)	87 (61.27%)	8.876 $\times \;10^{-7}$
dietary diversity for children aged between 4 and 6 months	21 (37.50%)	35 ( 62.50%)	0.02816
dietary diversity for children aged more than 6 months	80 (54.79%)	66 (45.20%)	0.02816
Factors related to early childhood			
Overweight during the first 2 years	11 (78.57%)	3 (21.43%)	0.02705
Consumption of antibiotics during first 2 years	32 (74.42%)	11 ( 25.58%)	0.0003174
Exposure to pollution in the first two years	14 (60.87%)	9 (39.13%)	0.2693

**Table 2:** Association of prenatal factors with childhood asthma using univariate logistic regression.

Variable	OR	2.5%	97.5%
Maternal atopy	19.04	3.83	126.39
Reported dust mites in the child’s environment	101.23	13.39	2271.27
Maternal age ≤ 25 years	7.19	1.81	33.17
Maternal age ≥ 35 years	53.13	4.24	850.82
Cold air in the child environment	21.62	2.18	335.19
Respiratory infections in family members (cold)	5.98	1.32	31.15
Respiratory infections in family members (flu)	11.61	2.31	76.33
Paternal age ≥ 34 years	13.50	2.66	84.79
Cesarean mode of birth	6.77	2.12	25.75
Breastfeeding in the first two years	0.03	0.01	0.12
Dietary diversity for children aged between 4 and 6 months	0.35	0.09	1.24

**Table 3:** Variable importance using a random forest model.

Variable	Mean Decrease Gini
Breastfeeding	9.49
Reported dust mites in the child’s environment	9.37
Maternal atopy	4.93
Cesarean mode of birth	4.18
Maternal age of ≤25 years	3.99
Antibiotic use during the first 2 years	3.59
Respiratory infections in family members (cold)	3.41
Paternal age of ≤25 years	2.85
Maternal obesity during pregnancy	2.05
Respiratory infections in family members (flu)	1.89
Consumption of antibiotics/paracetamol during pregnancy	1.77
Dietary diversity for children aged between 4 months and 6 months old	1.72
Dietary diversity for children aged more than 6 months	1.62
Cold airflow in the child environment	1.57
Strong odors in the child’s environment	1.39
Overweight in the first 2 years	1.27
Pollen in the child environment	1.14
Mold in the child environment	0.99
Maternal age of ≥35 years	0.82

**Table 4:** Performance comparison of different prediction models.

Performance Metrics	Logistic Regression	Decision Tree	Random Forest	SVM
F1 score (y = Asthmatic)	0.89	0.87	0.86	0.81
F1 score (y = Not Asthmatic)	0.83	0.82	0.89	0.80
Accuracy (%)	85.36	85.3	87.8	80
Average accuracy for 10-fold cross validation (%)	82.57	75.19	84.9	82.5
Sensitivity, Sn (%)	83	91	87	67
Specificity, Sp (%)	88	78	88	94
